# How Resilient Are Deep Learning Models in Medical Image Analysis? The Case of the Moment-Based Adversarial Attack (Mb-AdA)

**DOI:** 10.3390/biomedicines10102545

**Published:** 2022-10-12

**Authors:** Theodore V. Maliamanis, Kyriakos D. Apostolidis, George A. Papakostas

**Affiliations:** MLV Research Group, Department of Computer Science, International Hellenic University, 65404 Kavala, Greece

**Keywords:** adversarial attack, medical image analysis, computer vision, deep learning, adversarial training, robustness, image moments

## Abstract

In the past years, deep neural networks (DNNs) have become popular in many disciplines such as computer vision (CV). One of the most important challenges in the CV area is Medical Image Analysis (MIA). However, adversarial attacks (AdAs) have proven to be an important threat to vision systems by significantly reducing the performance of the models. This paper proposes a new black-box adversarial attack, which is based οn orthogonal image moments named Mb-AdA. Additionally, a corresponding defensive method of adversarial training using Mb-AdA adversarial examples is also investigated, with encouraging results. The proposed attack was applied in classification and segmentation tasks with six state-of-the-art Deep Learning (DL) models in X-ray, histopathology and nuclei cell images. The main advantage of Mb-AdA is that it does not destroy the structure of images like other attacks, as instead of adding noise it removes specific image information, which is critical for medical models’ decisions. The proposed attack is more effective than compared ones and achieved degradation up to 65% and 18% in terms of accuracy and IoU for classification and segmentation tasks, respectively, by also presenting relatively high SSIM. At the same time, it was proved that Mb-AdA adversarial examples can enhance the robustness of the model.

## 1. Introduction

Computer Vision has evolved dramatically with the introduction of Deep Learning (DL) models [1] as the accuracy and the effectiveness of deep convolutional neural networks [2] became impressive. DL has been applied with great success to nearly all CV tasks e.g., classification [2], semantic segmentation [3], object detection [4], pose estimation [5], quality assessment [6] and depth prediction [7]. The application of CV in Medical Image Analysis [8] is mostly referred to segmentation and classification problems. The application of CV to MIA compared to other CV applications, seems to have an advantage due to the fact that medical images are mostly ideally captured, lacking occlusion and miss-orientation problems and minimizing distortion, deformation and mis-illumination problems that other images generally present. Lately, the pandemic of COVID-19 showed that doctors and personnel can never be enough and that everything that can help them is valuable. This underlines the need for the implementation of computer vision-based automated medical image analysis in a safe way.

When Adversarial Attacks appeared on DL models [9], CV became seemingly unreliable. A great research field of Adversarial Computer Vision (AdCV) was born in order to create greater security in the models [10,11,12]. Driven by the success of adversarial attacks on natural images, researchers implement adversarial examples on medical images to investigate the MIA models’ robustness [13]. The majority of studies have been done on classification and segmentation tasks. Dermoscopy images were tested in [14,15] for classification tasks. MRIs have been tested on classification tasks [16,17] and segmentation tasks [18,19]. For X-ray images, researchers in [20,21] proved that general purpose attacks are able to importantly decrease the models’ accuracy. Moreover, attacks were implemented in fundoscopy images in [22,23] presenting high attack accuracy. The aforementioned studies used several attacks such as Fast Gradient Sign Method (FGSM), Projected Gradient Descent (PGD), DeepFool, Jacobian-based Saliency Map Attack (JSMA), Universal Adversarial Perturbations (UAPs), Basic Iterative Method (BIM) and Carlini & Wagner (C&W), which significantly reduced the MIA models’ accuracy.

Additionally, several custom medical imaging attacks were developed. In [24] created an attack for Ultrasound (US) images for classification tasks. This attack was tested on the InceptionResNetV2 model. The authors in [25] created an attack for segmentation on fundoscopy and dermoscopy images, using the U-Net model. In addition, [26] developed an attack for fundoscopy images, which can be implemented on segmentation and classification tasks. Kugler et al. [27] tried to lead five models into misclassification on dermoscopy images, (ResNet, InceptionV3, InceptionResNetV2, MobileNet, Xception). Vatian et al. [28] created an attack, which is based on the “natural” noise of medical imaging systems. Experiments were carried out on CT scans and brain MR images. Chen et al. [29] generate adversarial examples for medical image segmentation, which are tested on CT scans. Another interesting attack was created by Tian et al. [30] and it is based on a biased field phenomenon.

DL is more efficient than traditional techniques because it provides powerful models that can achieve very high accuracy in difficult medical problems just by retraining some pretrained models. That is why DL models should be resilient to adversarial attacks, as it is a critical research task that will help CV to become reliable. Trustworthiness together with the explainability of DL models, which means the ability to explain how and what features are modeled inside the DL model to extract a decision, are two qualities of the utmost importance to achieve the goal of integrating this technology into medical practice. The contribution of this work is to:

(1) Highlight the vulnerability of medical images to attacks and try to explain why some tasks are more vulnerable to attacks than others.
(2) Propose a generalized attack that significantly affects the operation of DL models.
(3) Investigate adversarial training using the proposed attack method as a universal defense method.

The proposed attack has the following features:

(1) Can be characterized as a black box attack since there is no need for any knowledge of the MIA task, the DL model structure, or the dataset used for its training.
(2) Has several degrees of freedom and can be adapted to any DL model and any image resolution.
(3) Its effect is adjustable.
(4) The way it affects DL models is fully explainable, adding clues to how we can get closer to the DL interpretability or explainability goal.

## 2. Materials and Methods

Image moments are image projections on a basis produced by monomials or polynomials [31]. The first set of moments introduced were “geometrical moments” that can be produced using the following equation:

(1) $$M_{pq}=\overset{+\infty }{\underset{-\infty }{\int }}\overset{+\infty }{\underset{-\infty }{\int }}x^{p}y^{q}f\left(x,y\right)\;dxdy$$

where *f*(*x*,*y*) is the density distribution function of a single image channel and *p*, *q* are integer numbers. For color images, the moment set consists of three sets one for each image channel produced using the same equation.

The uniqueness theorem presented by Hu [32] proves that a piecewise continuous and fractured function f(x,y), that doesn’t have infinite non-zero values, can be uniquely determined by a sequence of moments $\left\{M_{pq}\right\}$ and conversely. Digital images are represented by a density distribution function f(x,y), which obviously satisfies Hu theorem’s conditions, except continuation. Such a function is discrete and therefore not continuous, that is the reason why we need discrete polynomials to produce the basis. In the above means the Equation (1) for discrete polynomials can be written as:

(2) $$M_{pq}=NF\left(p,q\right)\overset{N}{\underset{i=1}{\sum }}\overset{M}{\underset{j=1}{\sum }}Kernel_{pq}\left(x_{i},y_{j}\right)f\left(x_{i},y_{j}\right)$$

where $Kernel_{pq}$ is the product of specific polynomials [33] and is the appropriate normalization factor for the polynomial family selected.

It can easily be concluded that the maximum order of the moments is equal to the maximum of the values of the image dimensions *N*, *M*, so the produced moment set has the same dimension as the image (N×M).

Any discrete polynomial set that can produce a basis can be used to produce moments, but orthogonal polynomial families are mostly used. The orthogonality property simply means that the moments production procedure can be easily inverted using the same products of polynomials used for moment production.

When we collect the sequence of moments $\left\{M_{pq}\right\}$, we can use them to reconstruct the image in a simple way, due to the orthogonality property of the polynomials used in $Kernel_{pq}$ (2), using the equation:

(3) $$\hat{f}\left(x,y\right)=\overset{K-1}{\underset{p=0}{\sum }}\overset{L-1}{\underset{q=0}{\sum }}Kernel_{pq}\left(x,y\right)M_{pq}$$

Noting that in (3) according to the uniqueness theorem, if the limits *K* and *L* are equal to image dimensions *N* and *M*, respectively, the estimated density distribution function $\hat{f}\left(x,y\right)$ is theoretically identical to the initial function f(x,y). Moment sets can fully describe and reconstruct the image, which explains why they were broadly used as image descriptors.

The explanation of moments in physics and mathematics shows that lower order geometrical moments carry the greatest amount of information and higher order moments carry details. In image moments, lower order geometric moments represent some well-known image properties such as the total mass, the center of mass, the orientation and many others. In addition, moment functions which are invariant to scaling, translation (positioning), rotation and reflection, are called “moment invariants” [34], and they are generated using lower order moments. Lower order moment features describe the image, while on the other hand higher order moments contain information that is relatively useless for the task of image classification [35]. The way that the image information is distributed among image moments depends on the polynomial family that is used for moment production and on the family of expanding image moments. Specifically in image tasks, for the reasons mentioned earlier, discrete orthogonal image moments [31] are used. Most representative families are the Tchebichef, Krawtchouk, dual Hahn and Racah [32]. It is worth mentioning that moment set’s production is a computationally “heavy” task that can have large approximation errors, especially when many orders are needed. Numerous computational strategies [33] have been proposed, to minimize approximation errors and ease computational load.

The exclusion of some moments $M_{pq}$ of specific orders used for reconstruction by excluding some values of *p* or *q* from the sums of the reconstruction Equation (3), or practically substituting the specific moment values to be excluded with zeros, concludes in the production of an approximation of the estimated density distribution function $\hat{f}\left(x,y\right)$. Obviously, a good approximation is obtained using the optimal moments, those that carry most of the image’s information.

The proposed adversarial attack that we call Moment based- Adversarial Attack (Mb-AdA) produces the adversarial examples as follows:

- The image is transformed in a moment set $\left\{M_{pq}\right\}$ using a discrete orthogonal image moments family.
- Excluding some moments of specific orders, the image is reconstructed producing an approximation.
- The product can be used as an adversarial example to attack every DL model.

Τhe motivation behind the proposed attack is based on the fact that by eliminating some moment orders (possibly non-robust features) participating in the reconstructed image (attacked) the model will fail to detect this information and consequently will not compute the desired features within its deep structure.

Mb-AdA can be applied to any image form or resolution. DL models have a standard input resolution and that is not obvious to an image analysis system (IAS) user, because usually the system internally converts the image to the model’s standard color space and resolution before feeding it to the model’s input. In that case, every Mb-AdA can be used, even if it is constructed for the attacked model’s input resolution or not. The effect of Mb-AdA remains after any conversion made in the image processing part of an IAS.

The above, together with the fact that the attack is based only on image features, makes the proposed attack to be applicable to any DL model, without the need for any knowledge of its structure or the dataset that was used for training. Moreover, the goal of the proposed attack is not to misclassify an image in a specific category. That is why Mb-AdA can be clearly characterized as an untargeted black box attack. 

One other characteristic of Mb-AdA is that its effect is adjustable. As previously explained, the lower the moment’s order is, the more significant information is carried, which is why the exclusion of the last ordered moments is preferred. Using a smaller subset of moments during image reconstruction can increase the attack’s effect but with the cost of decreased image quality. On the other hand, acquiring better image quality of the adversarial examples excluding a smaller set of the last ordered moments, leads to a less efficient attack.

Adversarial Attack methods usually add an inexplainable noise to images, that is optimized in a different way in every attack method. Mb-AdA is the only that excludes specific known features of the image that makes its effect on DL models fully explainable. Moreover, studying the effect of several ordered Mb-AdAs we can explain in which moment features, is the DL model trained to base its decisions.

Obviously, for preparing only a specific Mb-AdA there is no need actually to compute entire the moment sets of the images in the first step but only the chosen subset of the moments that will be used for the reconstruction, lowering the computational cost of the procedure.

The experiments following the proposed adversarial attack uses the Tchebichef image moments family, that are constructed using (2) and the image reconstruction can be done using (3). The used $Kernel_{pq}$ in both formulas is the product of *pth* and *qth* order of the scaled Tchebichef polynomials given in (4) below. The used normalization factor NF(p,q), is the inverse of the product of the squared norms ρ(p,N) given in (5).

(4) $${\tilde{t}}_{n}\left(x\right)=\left(1/b\left(n,N\right)\right)\sum_{i=0}^{n}\left[\left(\begin{array}{c} N-1-i\\ n-i \end{array}\right)\left(\begin{array}{c} n+i\\ n \end{array}\right)\left(\begin{array}{c} x\\ i \end{array}\right)\right]$$

(5) $$\rho \left(p,N\right)=\overset{N-1}{\underset{x=0}{\sum }}{\left[{\tilde{t}}_{p}\left(x\right)\right]}^{2}$$

where *n* is the order of the polynomial, *N* − 1 is the maximum order and equal to the corresponding dimension value of the image and b(n,N) is a scaling constant, usually set equal to *N^n^*.

For constructing *K*-th order MB-AdA for the following experiments we used the following algorithm:

- Read image dimensions *N*, *M*.
- Select attack order K≤min{N,M}
- Compute the polynomials ${\tilde{t}}_{n}\left(x\right)$ using (4) and add them to a matrix.
- Compute normalization factor NF(p,q) using (5) and add them to another matrix.
- Compute moment set $\left\{M_{pq}\right\}$ for every image channel using Equation (2) with the above matrices. Compute the $Kernel_{pq}\left(x_{i},y_{j}\right),$ in every iteration of the sum from 1 to the selected order *K*, using the appropriate values from the polynomial matrix and saving them in a new Kernel matrix.
- Reconstruct the image using the above moment set$\;\{M_{pq}\}$ and the saved Kernel matrix using (3).

It should be mentioned that the reason we do not compute the entire moment set in the above algorithm is that, as explained earlier, we exclude only the last ordered moments from the reconstruction procedure. So, their computation would be not only useless but also computationally expensive.

For measuring the quality of the reconstructed (attacked) image, mean structural similarity index measure (SSIM) [36,37] is used. SSIM is one of the most representative ways to measure the difference between two images as it is correlated with the quality and perception of the human visual system [38] and it is given by:

(6) $$\mathrm{SSIM}\left(x,y\right)={\left(l\left(x,y\right)\right)}^{\alpha }{\left(c\left(x,y\right)\right)}^{\beta }{\left(s\left(x,y\right)\right)}^{\gamma }$$

where α , β and γ are weights (usually set to 1) of the terms luminance (*l*), contrast (*c*) and structure (*s*), that are given below as functions of mean values $\mu_{A\;}and\;\mu_{B}$, variance $\sigma_{A\;}and\;\sigma_{B}$ and covariance $\sigma_{AB}$ respectively:

(7) $$l\left(x,y\right)=\frac{2\mu_{A}\mu_{B}+C_{1}}{\mu_{A}^{2}+\mu_{B}^{2}+C_{1}}\;$$

(8) $$c\left(x,y\right)=\frac{2\sigma_{A}\sigma_{B}+C_{2}}{\sigma_{A}^{2}+\sigma_{B}^{2}+C_{2}}$$

(9) $$s\left(x,y\right)=\frac{\sigma_{AB}+C_{3}}{\sigma_{A}\sigma_{B}+C_{3}}$$

where $C_{1},\;C_{2}\;and\;C_{3}\;$are small constants used to stabilize the metric for the case where the means and variances become very small.

## 3. Results

### 3.1. Attack Results

**Figure 1:**
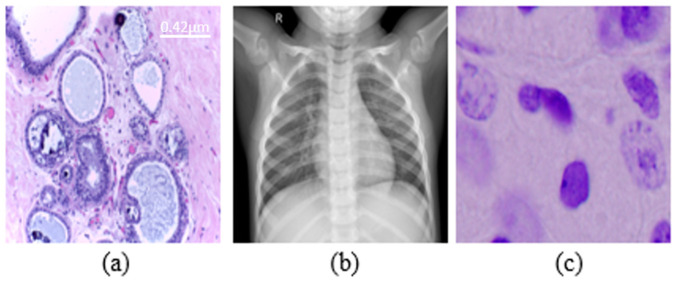
Examples from datasets were used. (**a**) Histopathology (scale bar—0.42 μm), (**b**) chest X-rays, (**c**) nuclei cells.

In order to measure the effectiveness of the proposed Mb-AdA to MIA tasks, we applied the attack, with several orders, in classification and segmentation tasks. The classification task was tested on X-ray [39] and histopathological [40] datasets. The first consists of three categories of lung X-rays (299 × 299 pixels size) while the second, which is more difficult, consists of four categories of cancer (2048 × 1536 pixels size). All datasets were divided into training, validation and test set. For the X-ray dataset, we used 3160 images for training 360 for validation and 365 for testing while in histopathological dataset we used 280/60/60 for training, validation and testing, respectively. These datasets, were used to train five DL models namely DenseNet 201 [41], Inception V3 [42], MobileNet V2 [43], DenseNet 169 [41] and Inception ResNet [44]. The segmentation task was tested on nuclei dataset [45] using the U-Net model [46]. For this task, we used 495 images (256 × 256 pixels size) for training and from 120 for training and validation. In Figure 1 examples of each dataset of the tasks are shown. The proposed attack was compared to FGSM [47], PGD [48] and Square Attack [49]. FGSM and PGD were trained in an irrelevant dataset in order for them to behave as black box attacks. All these attacks were created with Adversarial Robustness Toolbox (ART) [50]. All experiments were performed in Python with the Keras library. Additionally, we have experimented with several moment order values but for space saving reasons we present the most significant. As metrics we used Accuracy for classification task and Intersection over Union (IoU) for segmentation task.

$$Accuracy=\frac{True\;Positive+True\;Negative}{True\;Positive+True\;Negative+False\;Positive+False\;Negative}$$

$$IoU=\frac{Area\;of\;Intersection\;of\;two\;masks\;\left(predicted\;and\;ground\;truth\right)}{Area\;of\;Union\;of\;two\;masks\;\left(predicted\;and\;ground\;truth\right)}$$

The FGSM attack extracts the adversarial gradient and decreases or increases the value of pixels so that the loss function increases. It perturbs a clean sample for a one-step update along the direction of gradient descend. Projected Gradient Descent (PGD) attack like an Iterative FGSM. Perturbations are constrained by projecting adversarial samples from each iteration into L_∞_ or L_2_ neighbor of the clean image. Square Attack (SA) is based on a randomized search scheme which selects localized square shaped updates at random positions so that at each iteration the perturbation is situated approximately at the boundary of the feasible set.

In Table 1, nuclei segmentation results are presented with the SSIM (Structure Similarity Index Measure) and IoU (Intersection over Union) percentage that measures the accuracy of the segmentation for every attack. The indicating order number corresponds to the maximum order up to which is used to reconstruct the image (e.g., “Order 200” means that moments from 0 order up to 200 order, 201 × 201 moments in total are participating in the image reconstruction).

In Table 2 and Table 3, the results accuracy percentage for X-rays and histopathology images are presented, respectively.

### 3.2. Defence Results

The impact of Adversarial Training with Mb-AdA examples, augmenting the training dataset of the previously used ML models, as a defensive technique against adversarial attacks, is a reasonable question. Training DL models with images that lack non-robust features, as Mb-AdA examples are, should theoretically make them highly resistible to adversarial attacks. Numerous experiments were performed with several combinations of orders of images but for space saving, we will show indicatively the influence of adversarial training on MobileNetV2 with the histopathological dataset, which was the most vulnerable model. Table 4 shows the MobileNetV2 results with adversarial training using the initial training set and several moment orders.

## 4. Discussion

**Figure 2:**
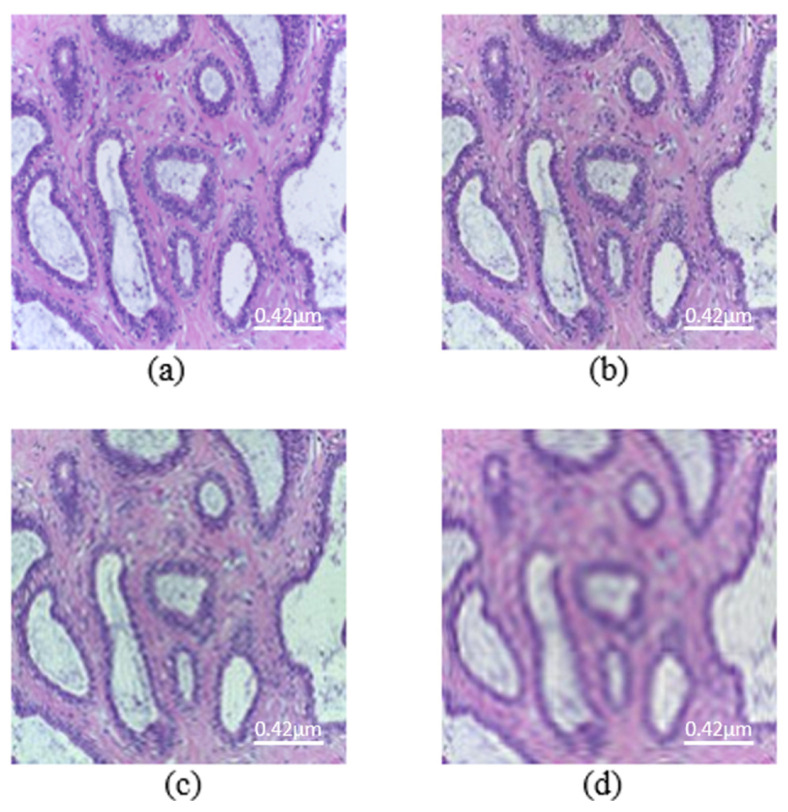
Images from the histopathological dataset (scale bar—0.42 μm). (**a**) Original image. Mb-Ada with order, (**b**) 200, (**c**) 120, (**d**) 50.

**Figure 3:**
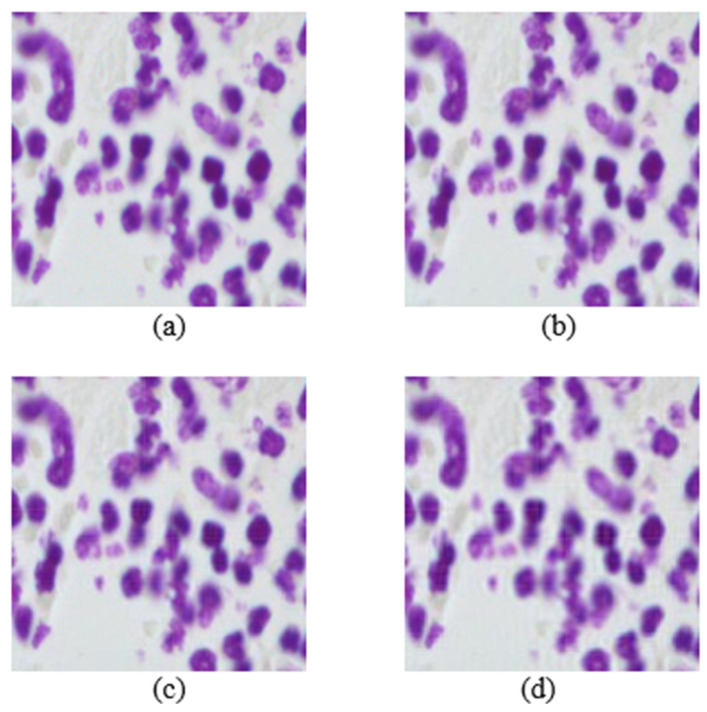
Images from the nuclei cell dataset. (**a**) Original image. Mb-Ada with order, (**b**) 200, (**c**) 120, (**d**) 80.

The main parameter of the proposed attack is the order of moments. Mb-AdA is an attack family that does not add some specific or optimized noise to an image as usually other adversarial attacks do, but it rather removes a part of the image. The removed part is not abstract but it consists of the last ordered moment features. It is worth noting that the first moment features carry the main image information, and the last ordered ones less. Figure 2 and Figure 3 are presented some examples of images under the proposed attack.

According to the results on X-ray dataset, Mb-AdA gets stronger as the order limit drops, because more and more of the higher ordered moment features are excluded, but as it is rational the image quality drops also. DenseNet 201, Inception V3 and Inception ResNet seem to be more resistant to higher order Mb-AdA than MobileNet V2 and DenseNet 169. 

Additionally, compared to the other attacks, Mb-AdA seems to keep higher the image quality when it starts to significantly drop the accuracy rates. For example, Mb-AdA order 80 drops the DenseNet 201’s accuracy score to 82.74 keeping SSIM to 91.35 when FGSM ϵ = 0.07 drops accuracy to the slightly worse 84.03 with SSIM to 76.35. PGD ϵ = 0.007 drops accuracy to the slightly worse 85 and also drops the SSIM to 77.87. The same happens with Square Attack ϵ = 0.07, which drops accuracy to 85.56 dropping the SSIM to 82.59, which is better than FGSM and PGD, nevertheless it can only be compared to Mb-AdA with order 40 and SSIM equals to 82 that drops accuracy score to 53.97. The proposed attack performs globally better classification than the other attacks in X-ray. On the other hand, the classification of histopathological images behaves differently. While SSIM drops, PGD, FGSM and Square Attack perform better than Mb-AdA. For example, Mb-AdA with order 100 and SSIM 66.88, achieved 71.67% accuracy while PGD ϵ = 0.3 with SSIM 65.32 and Square Attack ϵ = 0.2 with SSIM 65 achieved 53.33% and 61.67% accuracy, respectively. However, in high SSIM, the proposed attack outperforms the others attacks, which is our main goal. Τhe segmentation problem, strongly highlights the superiority of the proposed attack, as it globally shows a significantly greater reduction in terms of IoU compared to the other attacks, presenting a much higher image quality at the same time. The other attacks, achieved 3.5% IoU degradation even with very low SSIM, while our proposed attack achieved nearly 20% IoU degradation with significantly higher SSIM.

Mb-AdA is an attack that does not add some random or optimized noise to an image as usually other adversarial attacks do [13], but it rather removes a part of the image. The removed part is not abstract but it consists of the last ordered moment features. Removing from an image the last ordered non-robust features should have a small or no effect on DL models, since the image quality remains high. However, this effect is in most cases stronger than the other attacks. The reason can now be explained as the DL model during training, learning these non-robust features that are removed by the Mb-AdA, together with robust ones. The other attacks affect the non-robust and the robust features with the same weight factor, and that is why they importantly drop image quality in order to achieve the same scores with Mb-AdA. 

**Figure 4:**
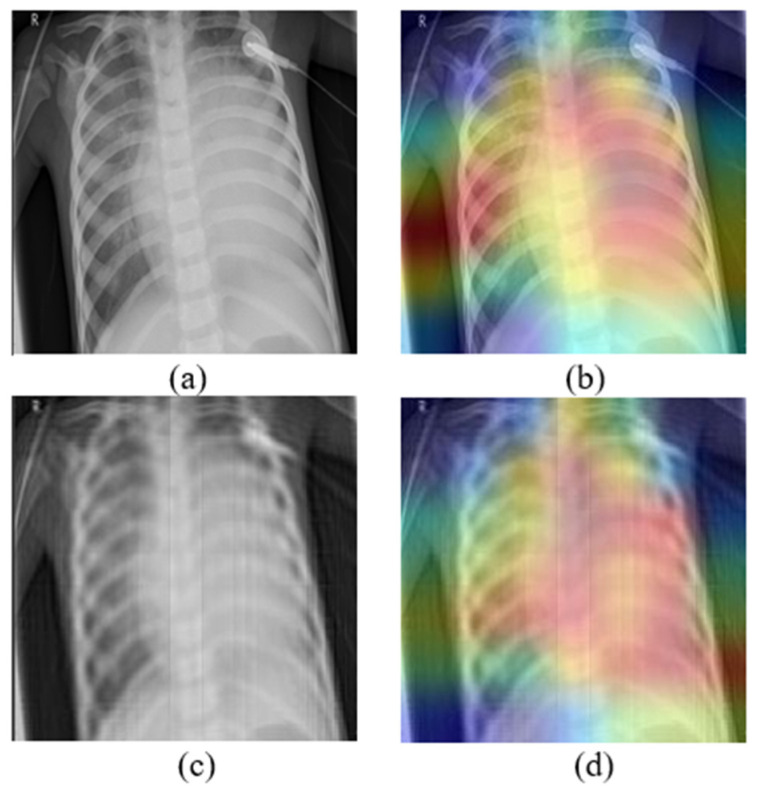
(**a**) Original image, (**b**) the corresponding GradCAM, (**c**) image under Mb-AdA with order 60, and (**d**) the corresponding GradCAM.

Moreover, medical image analysis tasks are quite difficult because models learn to make decisions according to some small details in the pictures. Mb-AdA has the ability to remove features that describe these details without destroying the structure of the image, and that is why it achieves high model’s performance degradation with high SSIM. The fact that the proposed attack does not destroy the structure of the image is also justified by the GradCAM algorithm [51] applied in Figure 4, which shows that models look at the almost same coordinates. This means that the attack preserves the structure of the images and does not disorient the model but removing useful information leads to misdiagnosis.

In addition to that, the data augmentation for adversarial training proves that it not only improves performance under Mb-AdA but improves performance even under other attacks. The MobileNetV2 model, which was the most vulnerable, has become quite robust after training with augmented data from the proposed attack in histopathological classification. The results are presented detailed in Table 4 but some representative examples are the following. The model trained with the Original Training Set (OTS) achieved 63.33% accuracy under FGSM ϵ = 0.03, while the same model trained with Augmented Training Set (ATS) achieved 90% accuracy. Additionally, the model with OTS under PGD ϵ = 0.1 achieved 60% accuracy while with ATS achieved 81.67%. These results come from the classification of histopathological images which was also the most difficult problem. It is clear that augmenting the data with examples of Mb-AdA significantly enhances the robustness of the model.

## 5. Conclusions

In this study, a black-box adversarial attack for medical images based on a moment exclusion strategy is proposed. The introduced attack was applied in classification and segmentation problems, achieving significant degradation in models’ performance while maintaining good image quality. Compared to other black-box attacks, the Mb-AdA shows better attacking results with additional improved imperceptibility. Additionally, adversarial learning was applied as a defense, proving that it can significantly enhance the robustness of models even under other attacks. The proposed attack can be useful for developing more robust deep learning models towards enforcing the integration of Artificial Intelligence tools in critical applications such as medical image analysis.

This work is sparking the future investigation into several directions, such as the optimization of the excluded moment orders per case, the incorporation of other moment families (e.g., Krawtchouk, dual Hahn, Fractional Moments, etc.) in the same attacking scheme and the examination of other medical image modalities.

## Figures and Tables

**Table 1:** Nuclei segmentation results under all attacks in terms of SSIM and IoU (in %).

ATTACK	SSIM	IoU
Attack Free	100	75.22
Mb-AdA Order 200	92.17	74.37
Mb-AdA Order 140	91.83	73.80
Mb-AdA Order 80	88.96	69.70
Mb-AdA Order 60	86.25	65.50
Mb-AdA Order 50	81.73	61.47
Mb-AdA Order 40	74.23	57.57
FGSM ϵ = 0.01	89.84	74.10
FGSM ϵ = 0.07	67.31	74.00
FGSM ϵ = 0.09	54.65	74.00
FGSM ϵ = 0.12	41.57	71.90
PGD ϵ = 0.01	89.73	74.12
PGD ϵ = 0.05	79.40	74.16
PGD ϵ = 0.07	70.45	74.15
PGD ϵ = 0.09	60.88	74.12
Square Attack ϵ = 0.01	90.14	74.00
Square Attack ϵ = 0.08	69.38	72.95
Square Attack ϵ = 0.1	65.00	72.28

**Table 2:** Image quality and accuracy in histopathological images under attacks (in %).

ATTACK	SSIM	DenseNet 201	Inception V3	MobileNet V2	DenseNet 169	Inception ResNet
Original	100	81.67	71.67	85.00	71.67	74.00
Mb-AdA Order 200	95.22	70.00	66.67	76.67	70.00	73.33
Mb-AdA Order 180	92.18	68.33	63.33	71.67	70.00	71.67
Mb-AdA Order 160	88.11	65.00	63.33	75.00	71.67	66.67
Mb-AdA Order 140	82.86	68.33	58.33	65.00	73.33	70.00
Mb-AdA Order 100	66.88	71.67	63.33	56.67	61.67	70.00
Mb-AdA Order 80	56.00	56.67	58.33	53.33	46.67	56.67
FGSM ϵ = 0.03	88.26	73.33	68.33	63.33	73.33	68.33
FGSM ϵ = 0.1	77.29	65.00	65.00	50.00	60.00	68.00
FGSM ϵ = 0.3	42.17	45.00	53.33	35.00	46.67	54.00
PGD ϵ = 0.01	89.46	75.00	66.67	76.67	75.00	76.67
PGD ϵ = 0.1	83.60	71.67	75.00	60.00	66.67	73.33
PGD ϵ = 0.2	65.32	63.33	58.33	40.00	55.00	66.67
PGD ϵ = 0.3	56.55	53.33	55.00	30.00	36.67	51.67
Square Attack ϵ = 0.01	89.70	76.67	70.00	81.67	73.33	73.33
Square Attack ϵ = 0.05	87.30	78.33	65.00	75.00	68.33	73.33
Square Attack ϵ = 0.1	80.90	66.67	56.67	60.00	65.00	75.00
Square Attack ϵ = 0.2	65.00	61.67	43.33	51.67	61.67	61.67

**Table 3:** Image quality and accuracy in chest X-ray images under attacks (in %).

ATTACK	SSIM	DenseNet 201	Inception V3	MobileNet V2	DenseNet 169	Inception ResNet
Original	100	99.18	93.97	97.81	97.26	97.26
Mb-AdA Order 200	99.03	98.90	93.42	91.51	93.42	96.99
Mb-AdA Order 160	98.13	98.90	92.05	87.67	94.52	96.44
Mb-AdA Order 120	95.26	96.90	89.04	80.55	89.59	94.25
Mb-AdA Order 80	91.35	82.74	81.10	56.16	69.32	86.58
Mb-AdA Order 50	85.43	60.82	59.73	43.84	36.70	61.37
Mb-AdA Order 40	82.00	53.97	40.55	42.47	33.15	60.00
Mb-AdA Order 30	77.89	40.55	34.52	40.27	32.88	54.25
FGSM ϵ = 0.01	98.71	98.36	91.23	91.78	93.42	95.34
FGSM ϵ = 0.03	94.60	96.40	84.93	80.27	88.49	92.60
FGSM ϵ = 0.05	84.10	90.14	73.15	53.15	70.41	84.11
FGSM ϵ = 0.07	76.35	84.03	66.11	42.30	68.35	76.75
FGSM ϵ = 0.09	64.23	73.11	57.14	39.78	64.71	69.75
PGD ϵ = 0.01	98.15	98.90	91.67	90.00	94.20	95.90
PGD ϵ = 0.03	93.84	96.40	83.30	75.56	82.70	87.50
PGD ϵ = 0.05	86.79	91.67	76.11	55.83	75.28	77.78
PGD ϵ = 0.07	77.87	85.00	65.56	49.44	68.61	70.56
PGD ϵ = 0.09	68.21	76.94	59.72	46.39	63.98	60.83
Square Attack ϵ = 0.01	99.29	98.61	91.94	91.94	93.61	96.67
Square Attack ϵ = 0.05	88.00	90.28	72.22	61.39	83.89	92.50
Square Attack ϵ = 0.07	82.59	85.56	58.06	55.83	77.50	91.39
Square Attack ϵ = 0.09	76.39	79.72	46.67	58.06	70.28	85.00

**Table 4:** Accuracy after adversarial training in histopathological images with MobileNetV2 (in %).

ATTACK	Normal Training Set	Augmented Orders 20–200	Augmented Orders 100–200
Original	85	88.33	81.6
Mb-AdA Order 200	76.67	90	73.33
Mb-AdA Order 180	71.67	90	71.67
Mb-AdA Order 160	75	86.67	75
Mb-AdA Order 140	65	85	73.33
Mb-AdA Order 120	65	88.33	76.67
Mb-AdA Order 110	65	85	80
Mb-AdA Order 100	56.67	86.67	75
Mb-AdA Order 80	53.33	81.67	76.67
Mb-AdA Order 60	45	70	63.33
Mb-AdA Order 50	45	68.33	68.33
FGSM ϵ = 0.03	63.33	90	76.67
FGSM ϵ = 0.05	60	88.33	58.33
FGSM ϵ = 0.1	50	71.67	61.67
PGD ϵ = 0.01	76.67	88.33	76.67
PGD ϵ = 0.1	60	81.67	70
PGD ϵ = 0.3	30	58.33	35
Square Attack ϵ = 0.02	80	86.67	85
Square Attack ϵ = 0.05	75	85	70
Square Attack ϵ = 0.1	60	68.33	75
Square Attack ϵ = 0.2	51.67	60	36.33

## Data Availability

Not applicable.
